# The Mutation Profile of SARS-CoV-2 Is Primarily Shaped by the Host Antiviral Defense

**DOI:** 10.3390/v13030394

**Published:** 2021-03-02

**Authors:** Cem Azgari, Zeynep Kilinc, Berk Turhan, Defne Circi, Ogun Adebali

**Affiliations:** Faculty of Engineering and Natural Sciences, Sabanci University, Istanbul 34956, Turkey; cemazgari@gmail.com (C.A.); zeynepkilinc@sabanciuniv.edu (Z.K.); berkturhan@sabanciuniv.edu (B.T.); defnecirci@sabanciuniv.edu (D.C.)

**Keywords:** SARS-CoV-2, COVID-19, evolution, mutation, phylogenetics, APOBEC, ROS, ZAP, ADAR

## Abstract

Understanding SARS-CoV-2 evolution is a fundamental effort in coping with the COVID-19 pandemic. The virus genomes have been broadly evolving due to the high number of infected hosts world-wide. Mutagenesis and selection are two inter-dependent mechanisms of virus diversification. However, which mechanisms contribute to the mutation profiles of SARS-CoV-2 remain under-explored. Here, we delineate the contribution of mutagenesis and selection to the genome diversity of SARS-CoV-2 isolates. We generated a comprehensive phylogenetic tree with representative genomes. Instead of counting mutations relative to the reference genome, we identified each mutation event at the nodes of the phylogenetic tree. With this approach, we obtained the mutation events that are independent of each other and generated the mutation profile of SARS-CoV-2 genomes. The results suggest that the heterogeneous mutation patterns are mainly reflections of host (i) antiviral mechanisms that are achieved through APOBEC, ADAR, and ZAP proteins, and (ii) probable adaptation against reactive oxygen species.

## 1. Introduction

The severe acute respiratory syndrome coronavirus 2 (SARS-CoV-2) has spread worldwide since its emergence in December 2019 [1], reportedly infecting more than 83 million people, with a death toll of 2,455,131 as of 22 February 2021, according to World Health Organization (WHO) (https://covid19.who.int/). Studies have been focused on effective treatment of the disease, mostly by the drug re-purposing approach due to the urgency [2] and by finding a vaccine that will stop the spread of the virus. Though there are dozens of vaccine candidates in clinical development, the evolutionary potential of the virus might affect the efficacy of the immunizations and treatments. Therefore, understanding the genomic features and mutation dynamics of the virus is crucial to interpret its evolutionary patterns and its response to the available treatments and potential vaccines.

Analyzing virus sequence context and mutations has revealed essential characteristics of SARS-CoV-2. For example, the origin of SARS-CoV-2 was linked to bats and pangolins using phylogenetic analyses [3,4,5,6]. Through mutational analyses, some genomic variants of the virus were associated with increased transmissibility [7,8]. In addition, we and others studied the spread of the virus in a variety of countries by tracking the mutation events of sequences over time [8,9,10].

Mutation profile analysis of SARS-CoV-2 can lead to the identification of mechanisms that drive the SARS-CoV-2 evolution; however, care should be taken when counting mutations to create a mutation profile. Considering that virus genomes are evolutionarily linked to each other, counting all the mutations in the sequences with respect to a reference genome creates a mutation bias towards the most abundant or frequently sequenced isolates. In other words, if a mutation occurs in an ancestral genome, it will also be seen in all of its descendants unless it reverts. When the mutations are called relative to a reference genome, variants of a common origin will be counted multiple times, even though they are linked to a single mutation event. To overcome this issue, we created a phylogenetic tree and assigned only nucleotides that differ from the parent node as a mutation. 

In this study, we retrieved SARS-CoV-2 genome sequences from the GISAID (Global Initiative on Sharing All Influenza Data) database [11] and analyzed the mutation profiles and sequence diversity of SARS-CoV-2. 

## 2. Methods

### 2.1. Data Retrieval and Mutation Assignment

495,159 SARS-CoV-2 genomes, their pre-computed multiple sequence alignment, and metadata in the GISAID database, which was dated until 9 February 2021, were retrieved [11]. Initially, the pre-computed multiple sequence alignment was used for filtering undesired genomes. The low-quality sequences (5% NNNNs) and duplicates were removed by providers of pre-computed multiple sequence alignment; we filtered out the genomes with more than (i) 30 single point substitutions; or (ii) 200 inserted nucleotides; or (iii) 200 deleted nucleotides (relative to the reference genome). Next, the remaining genomes (381,048) were obtained from the unaligned genome sequences for further analyses. Because alignment and tree construction with more than 380,000 genomes was computationally intense, the genomes were randomly subsampled to 30,000 with a custom bash script and all the sequences with incomplete information (proper date or location of sample collection) in the metadata file were filtered out with a custom python script. Then, we used cd-hit to cluster sequences and choose representatives (-c 0.9999 -M 0 -T 80) [12]. 18,050 clusters were created, of which 16,122 contained only a single sequence. Then, the first sequence of each cluster was assigned as the representative of that cluster. Representative sequences were aligned with the MAFFT algorithm using Augur toolkit [13,14]. Wuhan-Hu-1 genome (GenBank: NC_045512.2) was chosen as the reference genome for the alignment. Then, a phylogenetic tree was constructed using IQ-TREE (-fast -n AUTO -m GTR). 

The tree was then reconstructed into a time-resolved tree using the treetime option of Augur [13]. The sample with the earliest collection date among the representative sequences was chosen as the root, and marginal maximum likelihood estimation was used for date inference. The clock rate was applied across the genome to estimate the evolution rate and set to 0.0008, with a standard deviation of 0.0004 and using the date confidence flag to take the uncertainty of divergence time estimates into account. A constant coalescent model was chosen, and the “covariance-aware” mode of Augur was turned off with no covariance flag. 

To assign the mutations to the nodes of the time-resolved tree, the ancestral option of Augur, which infers the ancestral sequences, was used by giving the time-resolved tree and the multiple sequence alignment of representative sequences as input (inference joint). 

### 2.2. Mutation Profile Analysis

Mutation list was obtained from the phylogenetic tree and includes the mutations observed in each step of the tree. Then, this mutation list was divided into 192 groups based on their 12 mutation types (i.e., A > U, G > C) and 16 different trinucleotide contents where the mutating position is centered (i.e., A > U:UAA, G > C:AGU). Each of the 192 mutation groups were normalized with their corresponding trinucleotide count in the reference genome. Finally, these normalized mutation count values were plotted within the ggplot2 package [15] using R language, colored by their corresponding mutation type and trinucleotide content.

Observed mutations are first grouped by their position, mutation type, and trinucleotide, and frequently observed mutations (more than 8 times) at the same position in the same trinucleotide content are recorded. The observed mutations are grouped by their mutation type and trinucleotide, which resulted in 192 groups as indicated in the mutation profile. The contribution of each mutation to its profile is calculated, and the ones which contributed more than 10% are reported.

### 2.3. Measuring Codon Changes and Codon Usage

By using ancestral mutations from the time-resolved tree, the mutated codons (labelled as deformed) were counted, while the number of the forming codons were referred to as formed codons. For each codon type, the ratio between formed and deformed count was taken and plotted in log2 scale by using the ggplot2 package [15].

Human codon usage table was retrieved [16], SARS-CoV-2 codon usage table was calculated with a custom R script. Number of occurrences in the reference genome was retrieved for each codon, then, they were grouped by their corresponding amino acids. The ratio of use per codon was calculated by dividing the occurrence of that codon to the sum of itself and its synonymous codons occurrence. Afterwards, the relative ratio of codon usage between *Homo sapiens* and SARS-CoV-2 was calculated by dividing the ratio of a codon in one genome to the sum of ratios in genomes.

### 2.4. Dinucleotide Changes

Observed mutations in the time-resolved tree were used to calculate the number of deformations observed for each dinucleotide. Dinucleotides were formed by these mutations, which were also calculated and recorded as the number of formations. Then, observed counts in the reference genome per codon were retrieved. The deformation counts were normalized by their division with their observation counts in the reference genome and plotted with their formation counts by ggplot2 in R studio [15].

## 3. Results

**Figure 1:**
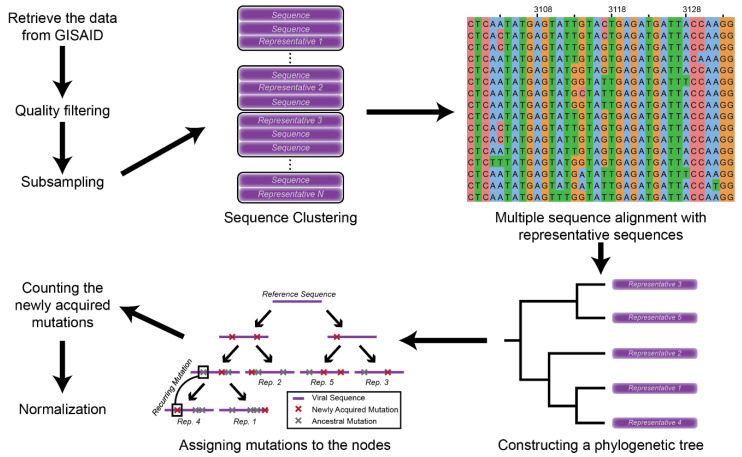
Schematic representation of the methodology.

We reconstructed a phylogenetic tree and inferred the ancestral sequences of the viral genomes [17]. After constructing the tree, mutations were assigned based on the differences between the sequences and their parent node (Figure 1). This method enabled us to capture all the mutation events without recounting ancestral mutations. Moreover, we could also identify mutations that occurred repeatedly in different lineages, which would not be possible if the mutations were assigned relative to a reference genome. 

### 3.1. Mutation Profile of SARS-CoV-2 and Potentially Related Mechanisms

**Figure 2:**
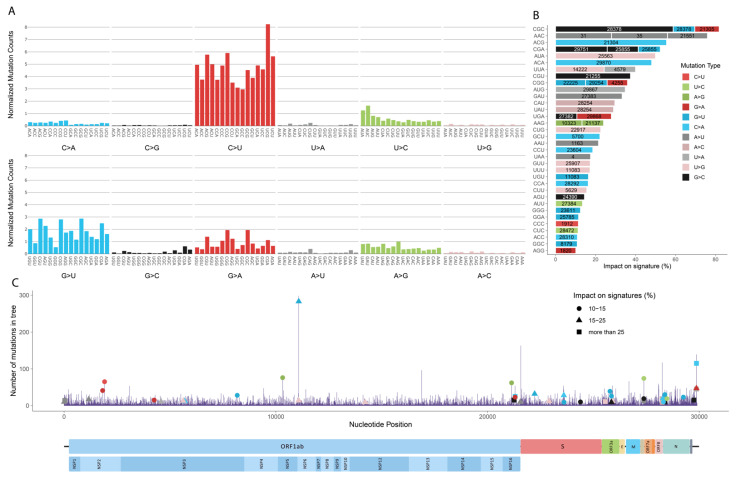
Mutation profile of SARS-CoV-2 genomes. Mutation counts are normalized by the trinucleotide content for each trinucleotide generated from 33,540 (representative sequences and nodes) sequences (**A**). From unstable positions where more than 8 mutations of the same type at the same position, highly occurred mutations are retrieved. The occurrences of these mutations are divided by the total number of the mutations of the same type and observed in the same trinucleotide. The calculated ratio is used to visualize the impact of highly occurring mutations on signature profile, as percentages (**B**). The bars are labelled with their positions in the genome. Number of mutations observed in the phylogenetic tree, per position (**C**). Mutations which have a significant contribution to their signature visualized in part B are marked according to the mutation count they represent at the position. Marked mutations are colored and reshaped with respect to their mutation type and impact range on their signatures.

To generate the mutation profile of SARS-CoV-2, we performed mutational signature analysis for all 192 trinucleotide changes using 54,353 mutations from the 33,540 representative sequences and nodes (Figure 2A). We normalized all the trinucleotide changes by the occurrence of the corresponding trinucleotide in the reference genome to eliminate any sequence context bias. In general, the most abundant mutational patterns are C > U, G > U, U > C, and A > G substitutions, that are 46%, 18.2%, 9.4%, and 8.8% of total substitutions, respectively (Figure 2A).

An enzyme family known for causing C > U substitution is called apolipoprotein B mRNA editing enzyme, catalytic polypeptide-like (APOBEC) family. Enzymes of APOBEC family have an antiviral activity against some RNA viruses including coronaviruses [18,19,20]. Briefly, they can deaminate cytosine to thymine (uracil in the RNA genome), which can either result in C > U substitution on single-stranded viral RNA (plus strand) or G > A reflection if the C > U substitution occurs on a complementary strand (minus strand). In agreement with previous studies, the impact of APOBEC is highly visible at C > U substitutions, while it is relatively low at G > A substitutions (7.2% of total substitutions) [21,22,23]. This result suggests an asymmetric activity for APOBEC enzymes in favor of single-stranded viral RNA. Because virus RNA is frequently present as the plus strand, we see the effect of the APOBEC activity majorly in the form of C > U substitution relative to G > A, which reflects APOBEC activity on the negative strand during RNA replication. Moreover, APOBEC proteins show target inclination towards 5′-[T/U]C-3′ and 5′–CC–3′ motifs while deamination of cytosine [24]. Target sequence preferences of APOBEC proteins are observed in our mutational profile, where 5 out of 7 highest normalized mutational counts on C > U distributes along 5′-UC-3′ and 5′–CC–3′ motifs (Figure 2A). It is also experimentally found that A1CF RNA editing cofactor, which is APOBEC1 complementation factor, is among the SARS-CoV-2 RNA binders [25] that strengthens the hypothesis of APOBEC proteins’ activity on the C > U substitutions.

The second most prevalent substitution is G > U, which might be associated with reactive oxygen species (ROS) in APOBEC-related manner. A recent study revealed that DNA damage response mediated by APOBEC3A (a member of APOBEC family) results in ROS production [26]. ROS can induce oxidative DNA damage, usually transforming guanine into 7,8-dihydro-8-oxo-20-deoxyguanine (oxoguanine), which can pair with adenine and lead to G > U substitution [27,28]. However, to date, there is no direct evidence of ROS-caused damage in the SARS-CoV-2 genome.

Another mechanism that can mutate the viral genome is adenosine deaminase acting on RNA (ADAR), which is an enzyme that mediates deamination of adenine to inosine (A > I) and later changes to guanine (A > G) [21]. A > G (plus strand) and U > C (minus strand) substitutions are observed at similar levels (8.8%, and 9.4% of total substitutions, respectively) (Figure 2A). ADAR targets dsRNA, and therefore, equivalent levels of ADAR activity are expected to be present at both strands [21]. The symmetric mutation profile for this pattern strongly suggests that ADAR working on replication RNA is effective in A > G and U > C substitutions.

In the context of trinucleotides, mutations dominantly occurred in U(C > U)G, C(C > U)G, A(C > U)G, U(C > U)U, and A(C > U)U (Figure 2A). Notably, 3 out of the 5 most frequently changed trinucleotides contain CG at their second and third positions. To examine whether these mutations were predominantly located at a single position in the viral genome or are distributed throughout the genome, we identified dynamic positions, where more than 8 recurring mutations were observed. Afterwards, we investigated the contribution of these trinucleotide positions to the mutation profile (Figure 2B,C). With some exceptions, most mutations in dynamic positions do not dominate the overall mutation profile. One of the exceptions is G(G > C)G mutations occurred at position 28,883, which correspond to the 66.6% of all mutations occurring on GGG. Although the percentage is high, the number of mutations occurring on GGG trinucleotides is only 9. Similarly G(A > U)C mutations at position 29,869 correspond to the 29.4% of all mutations occurring on GAC trinucleotide, but the number of mutations of GAC is as low as 17. However, when trinucleotides with a total mutation number exceeding 500 are considered, the position with the highest mutation becomes position 11,083 with U(G > U)U mutations, composing 16.5% of total mutations of UGU. In conclusion, the mutation profile is not dominated by the switching positions; a position bias on mutation distribution is only observable when the total number of mutations of a trinucleotide is low. Several mutations labeled as impactful on signatures (Figure 2B) have been investigated for their possible effect on the severity and transmissibility of the virus by various studies. The highest mutational position, 11,083, has been associated with the severity of the virus [29]. Together with 11,083, mutations at 23,403, 21,575, 28,881, and 28,883 positions [30,31,32,33] have been associated with significant indication towards selection. In particular, the D614G mutation on Spike protein is associated with the fitness of the virus by both computational and clinical studies [32,33,34]. 

### 3.2. Codon Usage of SARS-CoV-2 Differentiates in Favor of A and U Containing Codons

**Figure 3:**
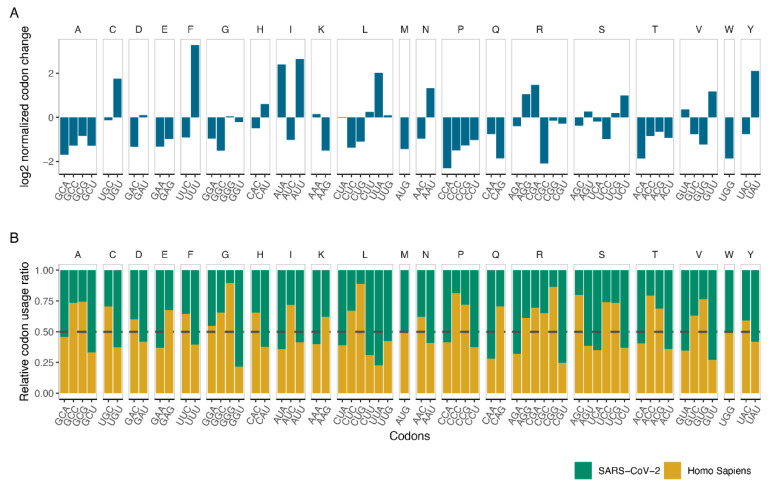
Comparison of codon variations in SARS-CoV-2 phylogenetic tree, human genome, and SARS-CoV-2reference genome. (**A**) Codon variations from mutations in the phylogenetic tree, represented by the ratio of formations over deformations per codon in log2 formation. (**B**) Relative codon usage percentage between Human and SARS-CoV-2 reference genome.

We investigated the impact of a potential contribution of codon bias selection on the mutation profile. First, we counted all the formed and altered codons, which we referred to as “form” and “deform”, respectively. We calculated the relative difference between form and deform values for each codon to test potential convergence of virus genome to host codon usage through mutations (Figure 3A). While UUU, AUA, AUU, and UAU are the intensively formed codons, CCA, UGG, GCU, and ACA are the most diminished ones. These results indicate a dominant forming of A and U containing trinucleotides, whereas G and C containing trinucleotides tend to reduce in number. In addition, all the codons that are translated into alanine (A) and proline (P) tend to diminish, resulting in lower translation of these amino acids in viral proteins. Considering that all the codons of A and P contain GC and CC in the first and second position, respectively, the reduction in these amino acids is probably related to selection against G and C presence (Figure 3A). 

Human coronaviruses are known to have low GC content (GC%), and SARS-CoV-2 is not an exception with ~38 GC% [35,36]. Moreover, it was suggested that the reduction in GC% is an adaptation strategy of SARS-CoV-2, particularly towards the codon usage of the genes expressed in the human lung [37]. To determine whether the mutations of SARS-CoV-2 is an adaptation strategy to increase its viability inside the host or just the byproduct of host immune response to the viral RNA, we obtained the human codon usage values [16] and calculated the codon usage of SARS-CoV-2 (see methods). We calculated the relative ratio of these values and grouped codons that are translating the same amino acid (Figure 3B). If the viral genome is to adapt to the host genome, one can hypothesize that the codons that are used dominantly in the host relative to the virus should be formed in the viral genome to increase the similarity, while the percentage of codons that are used dominantly in the virus should decrease. GCU, GAA, GGU, and CGU codons that are used relatively high in SARS-CoV-2, have the tendency to deform, in agreement with the hypothesis. However, UGU, AUA, UUA, and GUU codons that are also used relatively high in SARS-CoV-2 have the tendency to be formed. A similar contradiction is also observed in the codons that are highly used in the human genome. In general, adaptation to the host codon usage does not explain the formation tendency of the codons. The main driver of the formation tendency is likely to be selection pressure against GC%, and thus, A and U increase.

### 3.3. CG Nucleotide Deforms, While UU Nucleotide Forms

**Figure 4:**
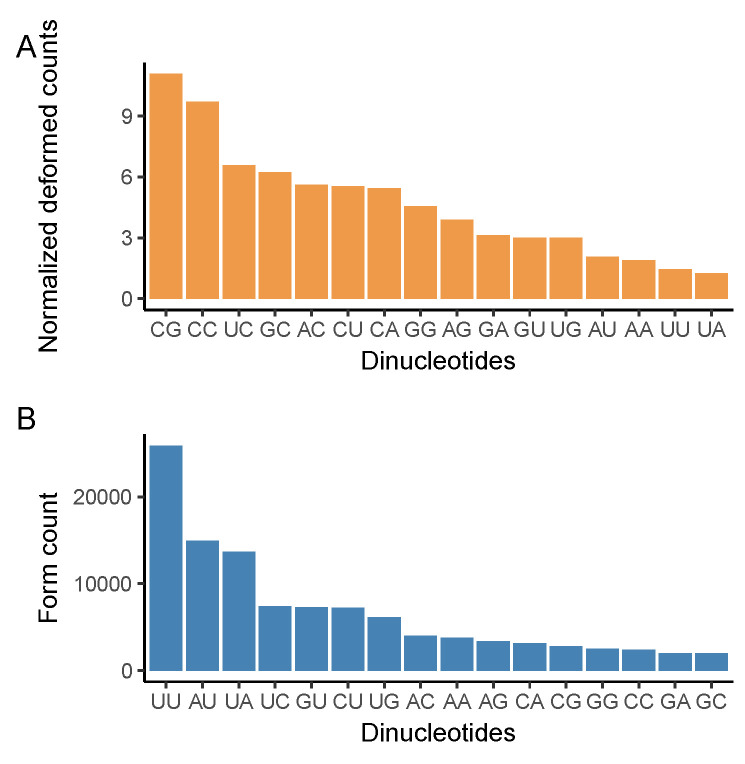
Comparison of dinucleotide formations and deformations retrieved from phylogenetic trees. Deformation ratio of dinucleotides is represented as the ratio of deformation count in the tree over dinucleotide’s abundance in the reference genome (**A**). As a result of the mutations, the relative dinucleotides are formed (**B**).

After observing excessive mutations in trinucleotides that contain CG at their second and third positions, and higher deformation in G and C containing codons, we examined the deformation (Figure 4A) and formation (Figure 4B) of dinucleotides. Because deformation of a dinucleotide is dependent on its occurrence in the genome, we normalized the deformed value of each dinucleotide with respect to its occurrence in the reference genome. As suggested by others [36,38], CG dinucleotide is the most deformed among all (Figure 4A). Xia et al. attributed the reduction in CG dinucleotide to a protein called zinc finger antiviral protein (ZAP), which binds and mediates the degradation of the viral genome [36]. This study indicates that SARS-CoV-2 is the most CG deficient betacoronavirus [36]. Thus, high CG deformation might be an adaptation of SARS-CoV-2 to escape ZAP under high purifying selection. In addition, UU dinucleotide is formed more than all other dinucleotides. In general, A and U containing dinucleotides are formed, meanwhile C and G containing dinucleotides are deformed.

## 4. Discussion

The COVID-19 pandemic has been spreading aggressively, killing thousands of people and affecting the daily lives of many more. Moreover, the evolutionary behavior of SARS-CoV-2 might potentially weaken the efficiency of the current treatments and vaccines. Here, we performed a phylogenetic tree-based mutational analysis to assess the contribution of mutagenesis and selection mechanism to SARS-CoV-2 mutation profiles. 

**Figure 5:**
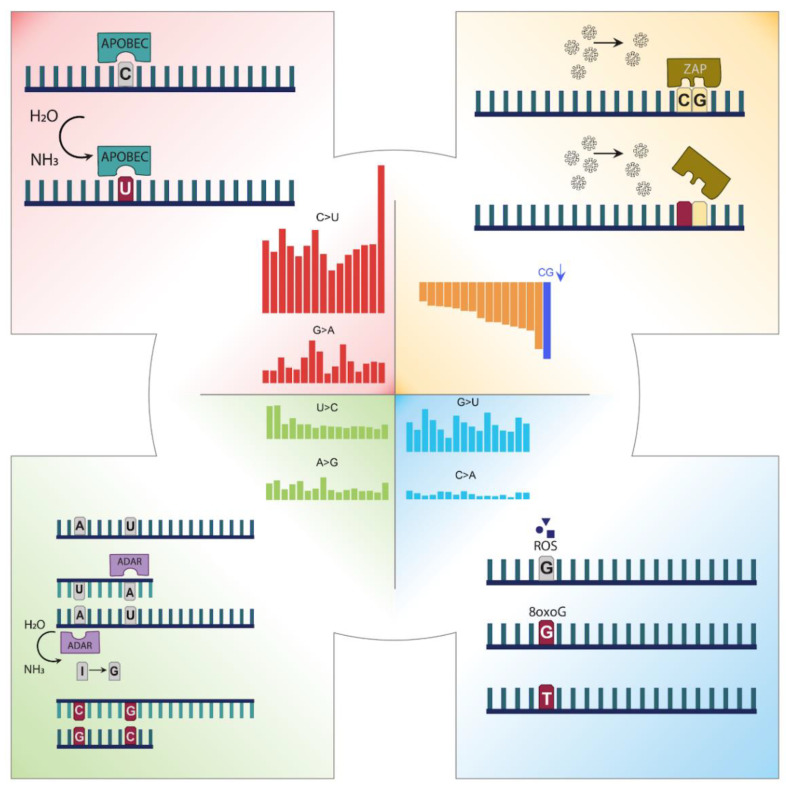
Mechanisms that can alter the sequence context of SARS-CoV-2. (i) APOBEC-caused mutations correlated with the enzyme signature dominantly on the plus RNA strand; (ii) ADAR-caused mutations equivalently affecting both RNA strands due to its mechanism of action; (iii) drop of CG dinucleotide targeted by ZAP through selection; (iv) ROS effect shown on the plus RNA strand.

The mutation profile of SARS-CoV-2 revealed that C > U, G > U, U > C, and A > G are the predominant substitutions. Based on these mutational patterns, we compiled some potential mechanisms that might be influencing the SARS-CoV-2 viral genome (Figure 5), which are namely APOBEC, ADAR, and ZAP. These mechanisms were linked to SARS-CoV-2 mutagenesis in previous studies as well [22,36,39,40]. In addition, we suspect that ROS might be a driver of G > U substitutions, however, more studies should be conducted to link ROS to SARS-CoV-2.

Another aim of this study was to examine the main driver of the mutational patterns of SARS-CoV-2; whether the viral genome is inclined to converge into the host genome or the mechanisms we have discussed are the only contributors to the mutational patterns. Analyses on formed and deformed codons exhibit an increase of A and U and a decrease of G and C containing codons. Furthermore, the comparison between human and SARS-CoV-2 codon usage does not reveal a strong correlation between codon usage percentages and SARS-CoV-2 formation tendency. These results combined suggest that SARS-CoV-2 genome diverges through RNA editing mechanisms of the host, independently of any adaptive mechanism to increase its genomic similarity to the host genome, which was suggested in another study as well [37]. Then, we examined the formation tendency of dinucleotides. In general, we observed a decrease of G and C, and an increase of A and U containing dinucleotides. Strikingly, the deform rate of CG dinucleotides and formation of UU dinucleotides are extremely high. This phenomenon, which was observed in most human viruses [41], was previously associated with the reduction of the hydrogen bonds between strands to achieve more efficient gene expression [38].

In conclusion, the mutational profile we generated supported the potential biological mechanisms contributing to the genome diversity of SARS-CoV-2 genomes. Strand asymmetry of some mutation signatures suggested the mechanism acting on the plus RNA strand only. Strand-wise equivalent mutation signature attributed to ADAR is in agreement with its mechanism of action where RNA is affected in the double-strand form. Antiviral responses and selection cannot be distinguished from each other. Host responses against the virus cause mutations in one hand, and the reduced targets in the virus genome make it less susceptible to the same antiviral attacks. Although we don’t suggest a direct antiviral mechanism to reduce CG content, the reduced CG content can be explained by an adaptation to the host antiviral mechanism by ZAP. So far, the virus has been affected by the host antiviral mechanisms. Although there are several Spike protein amino acid substitutions that are likely to provide a selection advantage [8,42], selection hasn’t been the major driving force of the genome-wide mutagenesis until the date of data collection. In the coming months, with a wide administration of the vaccines, it might be possible to see the effect of the vaccination and selection pressure by observing amino acid changes providing an advantage in escaping from immunized hosts. 

## Data Availability

All the codes and the processed data are publicly available at https://github.com/CompGenomeLab/SARS-CoV-2_Mutational_Profile.
